# Dosimetric effects of swelling or shrinking tissue during helical tomotherapy breast irradiation. A phantom study

**DOI:** 10.1120/jacmp.v15i4.4873

**Published:** 2014-07-08

**Authors:** Rudolf Klepper, Sebastian Höfel, Ulrike Botha, Peter Köhler, Felix Zwicker

**Affiliations:** ^1^ Klinik für Strahlentherapie/Radiologische Gemeinschaftspraxis Gesundheitsverbund Landkreis Konstanz Konstanz Germany; ^2^ Department of Radiation Oncology University Hospital Center Heidelberg Heidelberg Germany; ^3^ Clinical Cooperation Unit Molecular Radiation Oncology Deutsches Krebsforschungszentrum Heidelberg Germany

**Keywords:** breast, radiotherapy, swelling, shrinking, surface dose

## Abstract

During radiation therapy of the female breast, the actual target volume compared to the planning target volume may change due to swelling or shrinking of the tissue. Under‐ or overdosage is to be expected, especially when performing IMRT or tomotherapy techniques. The objective of this study is to develop a model‐based quantification of these dose effects, with a particular focus on the changes in the surface dose. A cylindrical phantom was used as an artificial surrogate of the human torso. By adding and removing Superflab layers of various thicknesses, both radial breast swelling and shrinking could be simulated. The effects on dose distribution were evaluated using film dosimetry. The results were compared to dose calculations. To estimate the true surface doses, we subtracted the influence of the film material on air measurements. During a swelling of 5, 10, and 15 mm, the planning target volume was consistently underdosed by 2%, 5%, and 7% of the prescribed dose, respectively. Swelling led to reduced dose values of up to 72%, 55%, and 50% at the outer edge of the actual target volume. The measured surface dose decreased successively from 31% to 23%. During shrinking, the dose in the planning target volume increased successively from 100% to 106%. The measured surface doses increased from 29% to 36%. The calculated dose values agreed with the measured values within error limits. During radiotherapy of the female breast, new planning appears to be essential for radial tissue swelling of 5 mm or more because of severe underdosing. Shrinking leads to moderate overdosing and an increased surface dose. In addition, caution is advised when removing bolus material with respect to the planned situation.

PACS numbers: 87.53.Bn, 87.55.dk, 87.55.D‐

## I. INTRODUCTION

Radiation therapy of the female breast has been expanded in recent years to include new techniques, such as intensity‐modulated radiotherapy (IMRT) and tomotherapy.^1^, ^2^ These new techniques offer improved precision, especially for dose homogeneity in the target volume and sparing of risk organs, such as the lungs and cardiac muscle.

However, there are some difficulties in exact positioning the target volume over all fractions.^3^, ^4^ Changes in dose are expected whenever the position of the target volume changes with respect to the planned situation. It has already been demonstrated that respiratory movement has an effect on the surface dose in helical breast irradiation.^5^, ^6^ Furthermore, it is known that volume changes, such as swelling of the female breast, are often followed by shrinking beyond the baseline status in the first two weeks of irradiation.^(3)^ The question of the present study is to what extent variations in breast size will affect the actual dose distribution when no adaptation of a plan or new planning is applied and when the daily image‐guided positioning is performed with standard matching of the chest wall. The extent of under‐ and overdosage is investigated.

As breast irradiation often causes skin reactions, such as radiation dermatitis (RTOG grade 1 or grade 2), special attention is given to the changes in the surface dose for different extents of swelling and shrinking. Measuring the surface dose is challenging, and the calculations at the surface should be interpreted with caution. In the literature, different outcomes are found when the calculated and measured surface doses are compared. Mutic and Low^(7)^ reported calculated values for the surface dose that were 20% higher than the measured values. Ramsey et al.^(8)^ published 3% to 13% higher calculated values and proved that the measurement differences are significant. Zibold et al.^(6)^ calculated surface doses that were 6% to 17% higher than the measured values. Capelle et al.^(9)^ reported an overestimate of calculated values of approximately14%, as did Tournel et al.^(10)^ In contrast, however, Akino et al.^(11)^ and Polednik et al.^(12)^ calculated dose values near the surface that were lower than the measurements.

The second aim of this publication is to minimize these discrepancies, and to ensure the calculated and measured values are consistent, thus leading to reliable estimations for the surface dose values.

## II. MATERIALS AND METHODS

The study was performed on a cylindrical phantom representing a female torso. Radial tissue swelling and shrinking were simulated by adding and removing bolus material, respectively. The treatment plan was created for a baseline situation. Dose calculations and measurements were performed for the baseline, as well as for the swollen/shrunken situations. The resulting dose profiles were compared.

### A. Material

**Figure 1:**
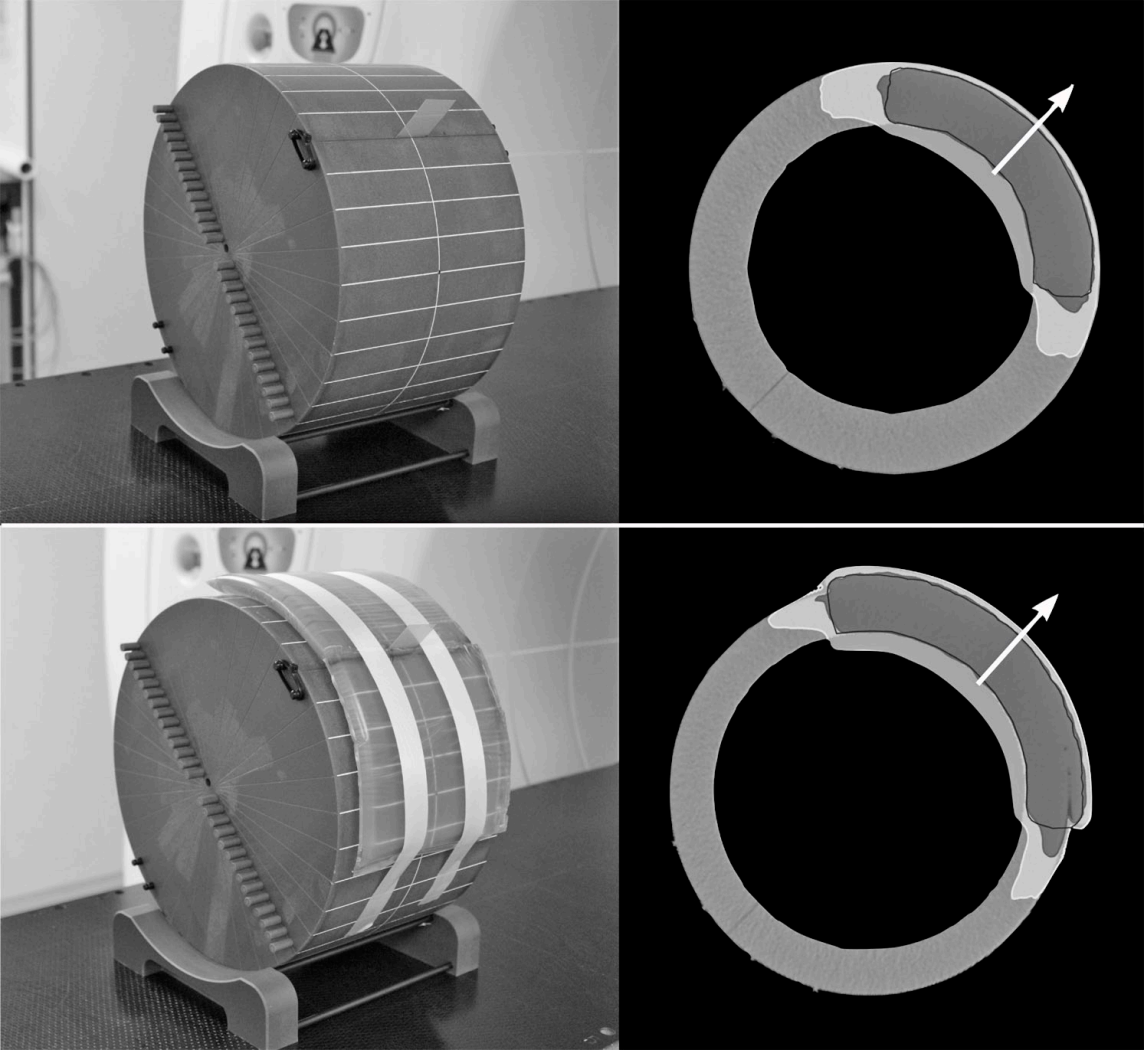
Simulation of the irradiation of the left breast. (Top) left: water‐equivalent cylinder phantom as the baseline medium for swelling, GAFCHROMIC film in a 45° position; right: dose plan cross section with PTV (black outline) and the OAR lung (black central area). The isodoses of the radiation plan are dark‐grey=5 Gy±5% and white ≥2.5 Gy<5 Gy. The dose profiles are investigated along the arrow. (Bottom) left: cylinder phantom as above, with a 15 mm Superflab layer as the baseline medium for shrinking, GAFCHROMIC film in a 45° position; right: dose plan cross section with PTV (black outline) and the OAR lung (black central area).

As an artificial surrogate for the human torso, a homogeneous cylindrical water‐equivalent “cheese‐phantom” (Accuray, Madison WI) was used. It can be separated lengthwise into two halves, thus enabling radial film dosimetry at any desired angle. The cylinder length was 180 mm, with a radius of 150 mm (Fig. 1). This radius roughly corresponds to the sum of the radius of a thoracic cavity and the radial thickness of a female breast. To simulate swelling and shrinking of breast tissue, we altered the radius of the phantom with body‐equivalent Superflab layers (Q‐Fix, Avondale, PA) (Fig. 1).

A 6 MV beam from a Tomotherapy Hi‐Art Accelerator (Accuray) was used for irradiation. Treatment planning was performed with the dedicated TomoTherapy treatment planning system (TPS) Version 4.2.1. The TPS uses a superposition/convolution algorithm with polyenergetic point kernels for dose calculations.^13^, ^14^

GAFCHROMIC EBT3 films (ISP, Wayne NJ) were used for film dosimetry, taking the recommendations of Schneider et al.^(15)^ into account (i.e., red channel extraction using the inner sector of the scanning field and scanning in landscape orientation). The films were cut into 3×10 cm strips. The exposed films were digitized using an Epson Perfection V750 PRO scanner (US Epson, Long Beach, CA) with the EPSON‐Scan software. A resolution of 300 dpi, 8‐bit gray levels, and no automatic image corrections were selected. Radial dose profiles were obtained by averaging the gray values in the middle centimeter of the 3 cm wide strip. Thereafter, the gray values were converted to dose values using calibration exposures taken immediately after the measurement series. The 1 cm wide edges of the films are used for pierce markings indicating the phantom surface.

### B. Procedure for swelling

The following procedure was applied to obtain a radiation plan representing irradiation of the left breast or chest wall, sparing the lung and cardiac muscle.

As in a supine patient position, the breast or thoracic wall geometrically approximates a ring segment; the planning target volume (PTV) was defined as a ring segment with a radial thickness of 42 mm. The PTV covers an arc from 355° to 95° within the cylinder and extends 6 cm in the longitudinal direction. There is a known overdosing of the skin with the standard customary IMRT calculation algorithms. This effect can be prevented by defining a skin‐saving layer of a few millimeters in thickness.^5^, ^16^, ^17^ Therefore, the outer edge of the PTV was drawn 4 mm below the surface. This is not in agreement with the guidelines of the RTOG, and is only recommended in cases with a sufficient distance between the skin and tumor bed. A central cylindrical volume was defined to represent the organ at risk (OAR) lung (Fig. 1) that reaches to the inner PTV edge. The middle of the phantom was placed at the isocenter of the tomotherapy unit. A helical tomotherapy plan was calculated using the following conditions.

A homogeneous distribution of a 5 Gy dose inside the PTV was prescribed, and maximum sparing of the lung was enforced by a directional block (i.e., prohibiting the passage of beamlets through the lung before entering the PTV). The plan calculation parameters in detail were: field width 2.5 cm, pitch 0.12, modulation factor 2.8, calculation grid $1.37\times 1.37\;{\text{mm}}^{2}$, and CT slice thickness 2 mm.

A representative 2D dose distribution resulting from this procedure is given in Fig. 1 (top).

To simulate swelling effects, the cylinder radius was increased step‐wise by 5, 10, and 15 mm, at each step adding Superflab material of a corresponding thickness onto the phantom surface. The added material was placed and affixed only over the region representing the left breast. This resulted in a three‐level breast swelling simulation. The actual target volume increased in the radial direction. The basic phantom and the expanded phantoms were called phantom0 to phantom15, according to the applied Superflab thicknesses.

Phantom0 and the expanded phantoms were exposed to a radiation plan, which was based on phantom0. The dosimetric effects for different extents of swelling could be measured via film dosimetry, and the resulting dose profiles were compared to the baseline situation (phantom0). For this purpose, GAFCHROMIC strips were inserted between the two phantom halves to a depth of 8 cm (Fig. 1). For the expanded phantoms, the strips were pushed through a slit in the Superflab material. Closure of the slit led to possible local changes in the layer thickness. The actual thickness could be determined from pierced markings on the film. For every film exposure, an absolute dose value at a depth of 20 mm in relation to phantom0 was measured with an ionization chamber. The measured curves were scaled in such a manner that the film dose values at a 20 mm depth corresponded to the absolute value in each case. All film exposures were made immediately after each other, including dose calibration.

To assess the changes in dose distribution, the integrated “Delivery Quality Assurance” tool (DQA) of the TomoTherapy software was used. The DQA tool offers the possibility of calculating the dose distribution in an arbitrary phantom resulting from the planned helical beamlet configuration. By using phantom0, as well as the expanded phantoms, calculated radial dose profiles could be extracted, which were then compared to the measured profiles.

### C. Procedure for shrinking

To investigate the effects of shrinking, a similar procedure was performed. The only difference was that the treatment planning was executed on phantom15. Here, the outer edge of the PTV was located 4 mm from the Superflab surface. The PTV thickness was kept at 42 mm. Thus, the radius of the lung cylinder increased accordingly by 15 mm (Fig. 1, bottom). Radial shrinking of −5, −10, and −15 mm was then simulated using phantoms phantom10, phantom5, and phantom0, respectively.

### D. Determination of surface doses

For surface dose calculations, the finest calculation grid ($1.37\times 1.37\;{\text{mm}}^{2}$) provided by the TPS was selected. Furthermore, a dedicated conversion table between the Hounsfield values and material density (IVDT table) was used, which influenced the calculated doses in the air and at the surface.^(18)^ This is due to the blurring effect of CT reconstruction algorithms at sharp material transitions. Thus, air voxels near the phantom surface had Hounsfield values higher than −1000 HU. The IVDT table was corrected in the low HU region as follows: −1024 HU was assigned to a density of $0\;{\text{mg/cm}}^{3}$, −1000 HU to $1\;{\text{mg/cm}}^{3}$, and −950 HU to $2\;{\text{mg/cm}}^{3}$.

Measuring the dose at the surface is challenging. The sharp material‐air transition calls for sensors of submillimeter extent. Basically, film dosimetry provides a high enough resolution to detect this transition sufficiently. However, the dose in air and at the surface is distorted because of the sensor material itself, which generates secondary electrons to a greater extent than air. Therefore, the true surface and air dose values are expected to be lower.

Using successive measurements with films of different thicknesses, it is possible to estimate the “sensor‐free” surface dose values; film piles containing 5, 3, or 1 film were exposed under identical conditions, as described above. Then, only the center film of each pile was evaluated. An additional dose calibration for low‐dose values led to “air‐dose” values for different film thicknesses. The extrapolation to thickness zero resulted in a dose value for the “sensor‐free” condition.

### E. General

To determine the effects of swelling and shrinking, the evaluation was restricted to radial dose profiles in the center of the PTV (i.e., along the 45° direction (arrows in Fig. 1)). A series of dose profile measurements at various angles (from 15° to 75° with a 10° increment) confirmed the uniformity among the profiles and, thus, permitted this restriction. Changes in the dose profile were only observed for the outermost angles ($>85^{\circ }$ and $<5^{\circ }$).

The thickness of the Superflab layer near the film deviated from its specified value by approximately +10% and required an exact measurement. The values are shown in Tables 1 and 2.

**Table 1:**
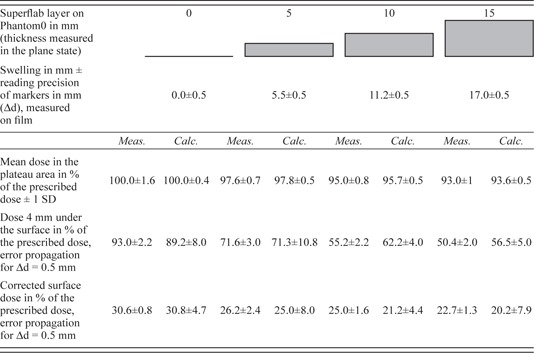
Results of the film measurements and calculations for swelling

**Table 2:**
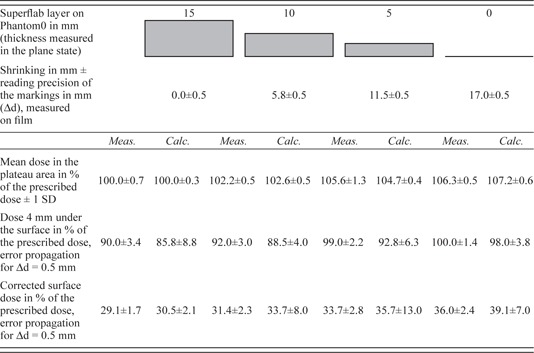
Results of the film measurements and calculations for shrinking

## III. RESULTS

### A. Dosimetric effects of swelling

**Figure 2:**
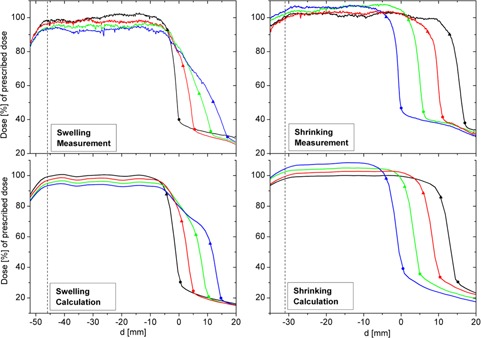
Measured and calculated dose profiles: black curves show the profiles in the baseline situation. The profiles for 5 mm, 10 mm, and 15 mm swelling or shrinking are colored red, green, and blue, respectively. The dotted line indicates the inner border of the PTVs. The outer borders of the target volumes and the phantom surfaces are marked with triangles and circles, respectively. (Top) left: measured profiles for swelling; right: measured profiles for shrinking. (Bottom) left: calculated profiles for swelling; right: calculated profiles for shrinking.

Figure 2 (left) shows the measured dose profiles along the 45° direction (see Fig. 1). The x‐axis represents the distance, d, to the surface of the baseline phantom (phantom0). The inner edge of the PTV is at d=−46 mm (dotted line). The black curve shows the dose profile for the baseline situation (i.e., no swelling). The curve contains a rising segment from d=−54 to −46 mm, where the PTV starts. It continues with a dose plateau to the end of the PTV at d=−4 mm. In the subsequent 4 mm thick layer between the PTV and the surface, the dose decreases sharply, which explains the desired skin‐sparing effect. Beyond the surface, the curve shape flattens off into the final section, which describes the measured dose in the air outside the phantom. The three other curves represent the corresponding profiles as measured for 5, 10, 15 mm of radial swelling.

The measured values of the actual Superflab thickness, the mean dose in the plateau, the dose value at 4 mm tissue depth, and the surface dose are listed in Table 1. The indicated uncertainties in the case of the “mean dose in the plateau” are a standard deviation of the measured values. Otherwise, they are to be understood as a propagation of measuring inaccuracy of the Superflab thickness Δd of 0.5 mm. For the calculations, the reading inaccuracy on the x‐axis is 1.37 mm, which corresponds to the resolution of the calculation matrix. The indicated precision limits for “surface dose” and “dose 4 mm under the skin” are defined as half of the difference between the calculated values of the two neighboring calculation points.

The calculated dose profiles display a qualitatively similar shape compared to the measured curves (Fig. 2). Within precision limits, the calculated curve parameters correspond to the parameters resulting from the measurements. In the case of the surface dose, accordance could be achieved only by applying the “sensor‐free” correction. The resulting curve parameters are given in Table 1.

### B. Dosimetric effects of shrinking

The measured and calculated dose profiles along the 45° direction are shown in Fig. 2 (right). The distance, d, is again defined in relation to the surface of phantom0. The resulting curve parameters are listed in Table 2. In addition, here, the measured and calculated values are also in accordance within precision limits. For the surface dose, accordance is only achieved using the “sensor free” correction.

## IV. DISCUSSION

### A. Swelling

The curves in Fig. 2 (left) and the low standard deviations for the dose values within the plateau area indicate that the dose distribution within the PTV remains relatively homogeneous. Furthermore, the width of the dose plateau remains unchanged. However, the average dose value decreases with increased swelling, for example, to approximately 93% of the prescribed dose at a Superflab thickness of 15 mm. This effect is expected because of the additional photon absorption in the applied Superflab material. The calculated values of the “mean dose in the plateau area” show an identical trend within error limits (Fig. 2 and Table 1).

In comparison, a conventional 3D irradiation with 6 MV tangential beams likewise reduces the mean dose in the PTV for 15 mm breast swelling by approximately 7% (calculation in tomo‐direct mode).

A 4 mm thick skin‐saving layer was included in the above‐described dose planning as a baseline (Fig. 2, top, left). In principle, the 4 mm layer is maintained in the various swellings, but the curves do not decrease from 93% of the intended dose to the surface dose values, but instead start to decrease from 71.6%, 55.2%, and 50.4%, respectively. A transitional layer of insufficient dose appears during swelling between the PTV and each skin‐sparing layer. This transitional layer has a thickness corresponding to the extent of swelling. The maximum underdosage in the swollen target volume is found right beneath the skin‐sparing layer and amounts to 28.4%, 44.8%, or 49.6% of the intended dose. Here, underdosage should be understood as the difference to 100% of the prescribed dose. These values are in good agreement with the calculated values of 28.7%, 37.8%, and 43.5% within error limits. The somewhat greater differences at 10 and 15 mm swelling can be explained by the Superflab layer, which is thickened by approximately 10% (see Table 1).

**Figure 3:**
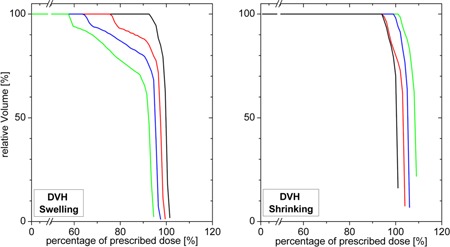
Dose‐volume histograms for the PTV: (left) swelling of 0, 5, 10, and 15 mm; (right) shrinking of 0, 5, 10, and 15 mm. Colors assigned as in Fig. 2.

The uniformity among the radial dose profiles through the PTV applies to a wide angle range, as well as for the adjacent CT slices. This is due to the simple geometry of the PTV in combination with the symmetric planning conditions. Thus, estimation for a common DVH representation (see Fig. 3) could be derived from the 45° dose profiles. Each of the estimated DVHs refers to the corresponding swollen or baseline target volume. The increasing underdosage with increasing swelling can clearly be observed.

The surface dose, determined by “sensor‐free” correction, decreases with increased swelling from 30.6% of the prescribed dose for zero swelling to 26.2%, 25.0%, and 22.7%, for each swelling thickness, respectively. These values are in good agreement with our calculated results. Without correction, the surface dose is 23.5% higher, which is in agreement with the results of Akino et al.^(11)^ and Polednik et al.^(12)^

### B. Shrinking

It is apparent from the curves in Fig. 2 (right) and from the results listed in Table 2 that the mean dose in the plateau area increases with increased shrinking from 100% to 106.3% of the prescribed dose. This effect occurs because of an increase in photon fluence in the reduced target volume as a result of missing absorptive tissue. The calculations indicate similar results (see Table 2). According to the guidelines given by ICRU Report 50, an overdosage inside the PTV of 106.3% is tolerable.

In an extreme case, with a shrinking of 15 mm, a conventional 3D irradiation with 6 MV tangential beams produces a dose increase to 107.5%, which approximately equals the value of the helical IMRT.

The 4 mm thick skin‐saving layer is maintained in the various degrees of shrinking, as well. The dose value at a 4 mm tissue depth increases with increasing shrinking from 90% to 100% of the prescribed dose. The calculated values correspond to the measured values within error limits. The greater differences in 10 and 15 mm shrinking can be explained by the Superflab layer, which is thickened by approximately 10%.

The estimated DVHs for shrinking (Fig. 3, right) show an increase of overdosage in accordance to the corresponding line profiles in Fig. 2 (right). There is no transitional layer as in the case of swelling.

The measured surface dose, determined by “sensor‐free” correction, increases with shrinking from 29.1% to 36% of the prescribed dose (Table 2) and is in good agreement with the calculated values. Shrinking leads to an increased surface dose and, therefore, intensifies skin erythema. In extreme cases of up to 15 mm radial shrinking, the surface dose may increase by approximately 7% of the prescribed dose.

## V. CONCLUSIONS

A severe underdosage is found in the case of swelling tissue in the swollen part of the target volume. An underdosage within the target volume of approximately 23% already occurs for 5 mm radial swelling.

Thus, our results suggest that, according to the recommendations of ICRU 50, a CT rescan and new planning should be considered for 5 mm of radial swelling. At greater swellings of more than 10 mm, a new plan is even more essential, as the underdosage in the swollen part of the target volume increases up to 50%, and even the region of the initial PTV is underdosed by more than 5%. If there is no requirement for skin protection, one can avoid this potential underdosing by adding bolus material during treatment planning and delivery.^19^, ^20^ However, caution is advised when removing the bolus material during treatment, as this can be observed as a “shrinking situation”. Although only a moderate overdosing of the target volume of up to 107% of the prescribed dose was noted in the case of shrinking, the increase in surface dose may be of concern.

Previous publications described discrepancies between the measured and calculated surface doses, most likely due to the influence of the sensor material. The extrapolation method was used to eliminate this influence. Within uncertainties, the results of film dosimetry and calculation were brought into accordance.

## Supporting information

Supplementary Material
