# Supplementary material for: Dosimetric effects of swelling or shrinking tissue during helical tomotherapy breast irradiation. A phantom study
Source: J Appl Clin Med Phys. 2014 Jul 8;15(4):382–91. doi: 10.1120/jacmp.v15i4.4873 (PMC5875514; doi:10.1120/jacmp.v15i4.4873)
Supplement: Supplementary file 1 — Supplementary Material [file ACM2-15-382-s001.doc]

**Dosimetric effects of swelling or shrinking tissue during helical tomotherapy breast irradiation. A phantom study.**

**Rudolf Klepper^1a^, Sebastian Höfel^1^, Ulrike Botha^1^, Peter Köhler^1^, Felix Zwicker^1,2,3^**

^1^ Klinik für Strahlentherapie im Gesundheitsverbund Landkreis Konstanz / Radiologische Gemeinschaftspraxis Konstanz, Germany

^2^Department of Radiation Oncology, University Hospital Center Heidelberg, Heidelberg, Germany,

^3^Clinical Cooperation Unit Molecular Radiation Oncology, Deutsches Krebsforschungszentrum, Heidelberg, Germany

All authors declare: There is no conflict of interest

**Short title:** Dose effects of breast swelling or shrinking

**Number of tables: 2**

# Number of figures: 3

a

**corresponding author:**

**Ph.D. Rudolf Klepper**

Klinik für Strahlentherapie im Gesundheitsverbund Landkreis Konstanz / Radiologische Gemeinschaftspraxis Konstanz, Germany

Haydnstrasse 2

D-78464 Konstanz.

klepper@radiologen-konstanz.de

*Tel.:* 0049 7531 8137122

*Fax:* 0049 7531 68626
